# Nb_2_O_5_ Microcolumns for Ethanol Sensing

**DOI:** 10.3390/s24061851

**Published:** 2024-03-14

**Authors:** Gayan W. C. Kumarage, Shasika A. Panamaldeniya, Valentin A. Maraloiu, Buddhika S. Dassanayake, Nanda Gunawardhana, Elisabetta Comini

**Affiliations:** 1 SENSOR Laboratory, Department of Information Engineering, University of Brescia, Via Valotti 9, 25133 Brescia, Italy; g.wadumasthree@unibs.it; 2 Department of Physics and Electronics, Faculty of Science, University of Kelaniya, Kelaniya 11600, Sri Lanka; 3 Postgraduate Institute of Science, University of Peradeniya, Peradeniya 20400, Sri Lanka; 4 Department of Physics, Faculty of Science, University of Peradeniya, Peradeniya 20400, Sri Lanka; 5 Laboratory of Atomic Structures and Defects in Advanced Materials, National Institute of Materials Physics, Atomistilor Str. 405 A, 077125 Magurele, Romania; maraloiu@infim.ro; 6 Research and International Affairs, Sri Lanka Technological Campus, Padukka 10500, Sri Lanka

**Keywords:** Nb_2_O_5_, microcolums: MOX, gas sensors, ethanol sensor

## Abstract

Pseudohexagonal Nb_2_O_5_ microcolumns spanning a size range of 50 to 610 nm were synthesized utilizing a cost-effective hydrothermal process (maintained at 180 °C for 30 min), followed by a subsequent calcination step at 500 °C for 3 h. Raman spectroscopy analysis unveiled three distinct reflection peaks at 220.04 cm^−1^, 602.01 cm^−1^, and 735.3 cm^−1^, indicative of the pseudohexagonal crystal lattice of Nb_2_O_5_. The HRTEM characterization confirmed the inter-lattice distance of 1.8 Å for the 110 plain and 3.17 Å for the 100 plain. The conductometry sensors were fabricated by drop-casting a dispersion of Nb_2_O_5_ microcolumns, in ethanol, on Pt electrodes. The fabricated sensors exhibited excellent selectivity in detecting C_2_H_5_OH (ΔG/G = 2.51 for 10 ppm C_2_H_5_OH) when compared to a variety of tested gases, including CO, CO_2_, NO_2_, H_2_, H_2_S, and C_3_H_6_O. The optimal operating temperature for this selective detection was determined to be 500 °C in a dry air environment. Moreover, the sensors demonstrated exceptional repeatability over the course of three testing cycles and displayed strong humidity resistance, even when exposed to 90% relative humidity. This excellent humidity resistance gas sensing property can be attributed to their nanoporous nature and elevated operating temperature.

## 1. Introduction

Nb_2_O_5_ gas sensors exhibit promising potential for multifarious applications encompassing environmental monitoring, industrial processes, and automotive domains. Notably, extensive investigations by researchers have explored the efficacy of Nb_2_O_5_ gas sensors in detecting H_2_S [1,2], H_2_ [3,4], NOx [5,6], CO [7], and VOCs [8,9] in environmental monitoring applications. 

Ethanol has been found in broad applications across various fields, including food safety, biomedicine, and fuel processing [10,11], that could potentially pose safety hazards if undetected. Its flammability and capacity for explosiveness in specific concentrations warrant prompt and accurate detection of any ethanol leaks or spills to avert potential risks. The integration of ethanol gas sensors plays a crucial role in the implementation of appropriate safety measures. By detecting the presence of ethanol in the air, these sensors can alert workers to impending dangers, safeguarding them from potential adverse effects such as irritations of the eyes, nose, and throat, as well as inducing headaches, dizziness, and even unconsciousness [12]. Consequently, ethanol gas sensing has emerged as a subject of utmost significance, garnering considerable attention in recent years, as evidenced by numerous scholarly works [13,14,15,16,17,18]. 

Metal oxides serve as fundamental elements in the evolution of gas sensor technology, with a significant emphasis on ethanol detection, which is attributable to their intrinsic characteristics and surface reactivity, as extensively documented in the literature [11,14,16,19,20]. Moreover, gas sensors based on metal oxides offer a plethora of advantages, encompassing cost-effectiveness, inherent stability, and compatibility with microfabrication methodologies, rendering them indispensable across a spectrum of sectors encompassing industrial, environmental, and medical domains. Their utilization spans from breath alcohol analysis to industrial process supervision and automotive emission regulation systems. As research endeavors progress, the fine-tuning of metal oxide attributes and sensor architectures presents a promising avenue towards elevating the sensitivity, selectivity, and dependability of gas detection mechanisms, thereby catering to exigent requisites in safety, healthcare, and environmental surveillance. The continued exploration in this domain holds the potential to revolutionize gas sensing technologies, advancing societal well-being and environmental sustainability.

Nb_2_O_5_, characterized as a typical n-type metal oxide, has garnered significant research interest in ethanol gas detection due to its distinct physical and chemical properties. In a recent scientific study, Mozalev et al. presented a novel 3-D niobium-oxide nanofilm, featuring a 200 nm thick NbO_2_ layer adorned with upright-standing Nb_2_O_5_ nanocolumns, each with a diameter of approximately 50 nm and a length of 900 nm. This sensor was fabricated using a porous-anodic-alumina-assisted anodizing technique of a refractory metal. It demonstrated remarkable capabilities in detecting 500 ppm of C_2_H_5_OH within a mere one-minute timeframe [21]. Furthermore, Khatoon et al. applied facile hydrothermal and solid-state methods to synthesize an alpha-Fe_2_O_3_(nanorods)/Nb_2_O_5_(nanoparticles) heterostructure for the detection of 100 ppm C_2_H_5_OH at an elevated temperature of 160 °C. The sensor exhibited a rapid response time of 8 s and a recovery time of 2 s, marking a significant advancement in ethanol sensing technology [22]. In another development, Lombardo and colleagues introduced Nb_2_O_5_ thin-film-based sensors designed to estimate blood ethanol levels. These sensors operated effectively at a relatively high temperature of 350 °C, contributing to potential applications in the medical field [23]. Additionally, Park et al. reported the creation of Pd and Au-functionalized Nb_2_O_5_ nanorods through thermal oxidation. These functionalized nanorods exhibited excellent performance in detecting 200 ppm C_2_H_5_OH at an operational temperature of 200 °C, with a noteworthy response rate of 183.54 (Ra/Rg%) [24].

One of the critical factors that affect the performance of Nb_2_O_5_ gas sensors is their sensing mechanism. The sensing mechanism of Nb_2_O_5_ gas sensors is based on the principle of metal oxide gas sensing, which involves the interaction between gas molecules and the surface of the sensing material. The adsorption of gas molecules on the surface of the sensing material can cause a change in its electrical conductivity. Another critical factor in the development of Nb_2_O_5_ gas sensors is the synthesis method used to prepare the sensing material. Researchers have investigated various synthesis methods, including hydrothermal [2,3,4] and sol–gel [25,26,27]-assisted methods, to prepare Nb_2_O_5_ gas sensors with different morphologies and properties. 

The present study elucidates the synthesis process of pseudohexagonal Nb_2_O_5_ microcolumns through a low-cost hydrothermal method, emphasizing their remarkable conductometric characteristics for ethanol detection down to sub-parts per billion (ppb) levels. The investigation reveals that the synthesized Nb_2_O_5_ microcolumns exhibit exceptional sensitivity and selectivity towards ethanol, owing to their unique structural and compositional properties. Notably, the sensors demonstrate negligible response fluctuations even under high relative humidity conditions of up to 90%, underscoring their robustness and reliability in real-world applications. The observed stability and high sensitivity of the Nb_2_O_5_ microcolumns suggest their potential for integration into advanced gas sensing platforms for precise and reliable ethanol detection in diverse fields, including automotive, industrial, and environmental monitoring. Moreover, the insights gained from this study contribute to the ongoing efforts to develop efficient and cost-effective gas sensing technologies for addressing critical societal and environmental challenges.

## 2. Materials and Methods

Nb_2_O_5_ microcolumns were synthesized using a specific procedure. Initially, commercially available Nb_2_O_5_ micro powder with a particle size of −325 mesh and a purity of 99.9% trace metals basis from Sigma Aldrich, Saint Louis, MO, USA was employed as the starting material. To synthesize the Nb_2_O_5_ microcolumns, 1.000 g of the commercial Nb_2_O_5_ powder was combined with 64 mL of a 10 mol dm^−3^ Sodium Hydroxide (NaOH) solution, which was of analytical-reagent grade (AR) and had a purity of 98%, obtained from Techno Pharmchem Haryana, Rohtak, India. The mixture was stirred for 30 min, and subsequently subjected to ultrasonication for 15 min. This stirring and ultrasonication process was repeated four times.

Next, the resulting mixture was transferred to an 80 mL autoclave and heated at 180 °C for a duration of 30 min. After this step, the formed precipitate was collected and subjected to a washing procedure. The washing process involved using a total of 1.2 L of a 1 mol dm^−3^ acetic acid (CH_3_COOH, Saint Louis, MO, USA) solution in increments of 300 mL each time. This was followed by another washing step with a total volume of 3.2 L of deionized water (DI) with a measured conductivity of 0.055 µS cm^−1^, performed in increments of 400 mL.

The basic chemical reactions during the Nb_2_O_5_ microwires growth can be written as follows [28].

The reaction starts with the dissolving of Nb_2_O_5_, in the presence of NaOH.

(1) $${Nb}_{2}O_{5(s)}+{2NaOH}_{\left(aq\right)}\to {2NaNbO}_{3(s)}+H_{2}O_{\left(l\right)}$$

During the CH_3_COOH washing, due to ion exchange;

(2) $${NaNbO}_{3\left(s\right)}+{CH}_{3}{COOH}_{\left(aq\right)}\to {HNbO}_{3(s)}+{CH}_{3}{COO}^{-}{Na}_{\left(aq\right)}^{+}$$

In the calcination process (700 °C), HNbO_3_ will convert to Nb_2_O_5_.

(3) $${2HNbO}_{3(s)}\to {Nb}_{2}O_{5(s)}+H_{2}O_{\left(g\right)}$$

Subsequently, the washed product was dried in a vacuum oven at 60 °C for a duration of 24 h and given a designation. Finally, the as-synthesized Nb_2_O_5_ microcolumns were subjected to a calcination process at 500 °C for 3 h, which resulted in the formation of the pseudohexagonal phase.

The Nb_2_O_5_ microcolumns obtained in this study were characterized by using X-ray diffraction (XRD) with a PANalytical diffractometer (Empyrean, PANalytical, Almelo, The Netherlands) equipped with a monochromatic CuKα beam (λ = 1.54184 Å). The measurements were conducted with a 2° incident angle, and the tube operated at 40 kV with a current of 40 mA. The diffraction pattern was scanned in steps of 0.05° with a step time of 25 s, covering the 2θ range of 20–80°. To examine the morphological properties of the prepared materials, a field emission scanning electron microscope (FE-SEM, model TESCAN MIRA 3, Brno, Czech Republic) was employed. Additionally, an analytical transmission electron microscope (TEM, JEM ARM 200F, JEOL, Munich, Germany) operated at 200 kV and equipped with a JED-2300T (JEOL, Munich, Germany) unit for Energy Dispersive X-ray (EDX) spectra was used for further analysis. For the TEM and EDX measurement, the Nb_2_O_5_ microcolumn powder was gently crushed in a mortar and diluted with ethanol. Next, the solution was sonicated for 5 min, and a drop of suspension was deposited on a copper grid with lacey carbon support film. 

The conductometric measurements were conducted within a custom-designed climatic gas chamber. A constant flow of 200 standard cubic centimeters per minute (sccm) of different gases, namely hydrogen (H_2_), ethanol (C_2_H_5_OH), acetone (C_3_H_6_O), nitrogen dioxide (NO_2_), hydrogen sulfide (H_2_S), carbon dioxide (CO_2_), and carbon monoxide (CO), was maintained during the experiments. The sensing elements were subjected to a fixed voltage of 1 V throughout the measurements.

To evaluate the sensor’s response to n-type behavior, the conductance of the sensor was compared under two conditions: firstly in the presence of the target gas, and secondly in synthetic air. The sensor’s response was quantified using the equation S (∆G/G) = (G_g_ − G_a_)/Ga for reducing gases or (G_a_ − G_g_)/G_g_ for oxidizing gases, where G_a_ represents the conductance of the sensor in synthetic air and G_g_ corresponds to the conductance of the sensor when exposed to the analyte gas [18,29]. This approach allowed for the characterization of the sensor’s performance in detecting and distinguishing between different gases, providing valuable insights into its sensitivity and selectivity.

## 3. Results

### 3.1. Material Characterization

**Figure 1:**
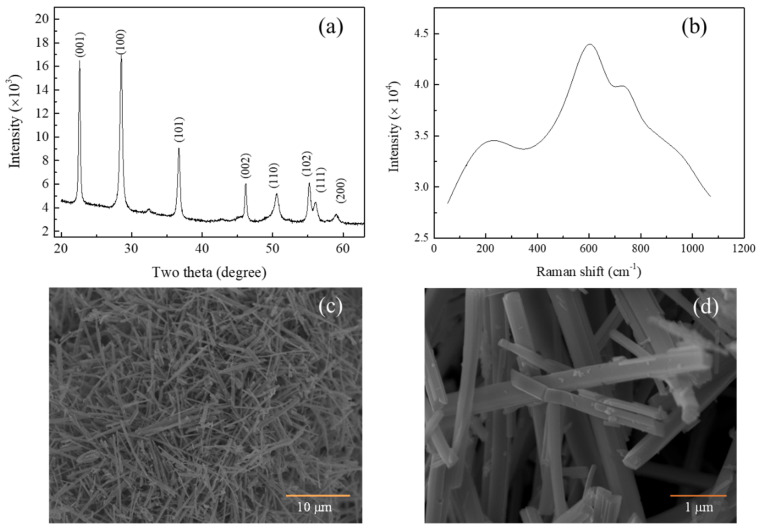
(**a**) XRD diffractogram; (**b**) Raman spectra; (**c**) FE–SEM of before the annealing; (**d**) FE–SEM of the synthesis Nb_2_O_5_ microcolumns.

The X-ray diffractogram of the synthesized Nb_2_O_5_ is presented in Figure 1a. The observed two theta reflection peaks align with the pseudohexagonal crystal structure characteristic of Nb_2_O_5_ (JCPDS No. 00-028-0317). Moreover, Figure S1 provides evidence that the synthesized material comprises solely Nb and O elements. In Figure 1b, the Raman spectra reveal three distinct reflection peaks located at 220.04 cm^−1^, 602.01 cm^−1^, and 735.3 cm^−1^, which correspond to the pseudohexagonal crystal structure of Nb_2_O_5_ [30].

The field emission scanning electron microscope (FE-SEM) image shown in Figure 1c depicts the intriguing morphological evolution of Nb_2_O_5_ micro powder (particle size less than 45 µm, −325 mesh size) during the hydrothermal reaction at a temperature of 180 °C. As the hydrothermal reaction proceeded for 30 min, the result was the formation of high-purity niobate microcolumns. The final product exhibits Nb_2_O_5_ microcolumns with dimensions on the order of tens of micrometers in length, hundreds of nanometers in width, and hundreds of nanometers in height, as illustrated in Figure 1d.

These results offer valuable insights into the structural and morphological characteristics of the synthesized Nb_2_O_5_ microcolumns, contributing to the understanding of their properties and potential applications in various fields.

**Figure 2:**
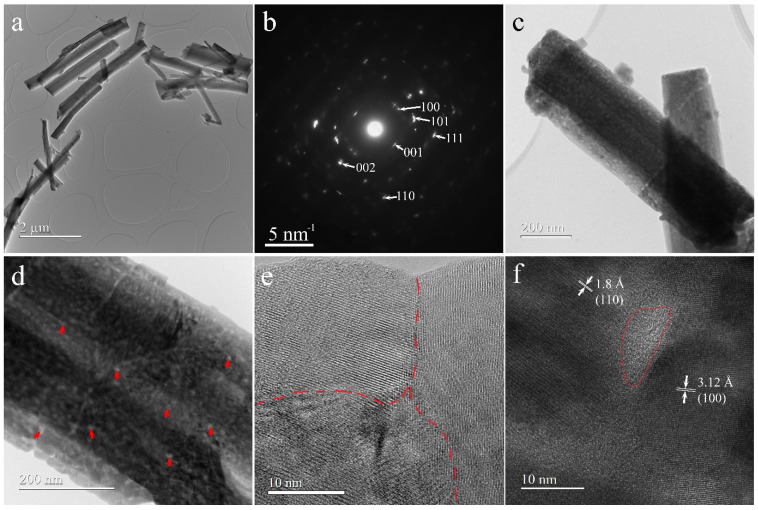
(**a**,**c**,**d**) CTEM images; (**b**) SAED pattern; (**e**,**f**) HRTEM images of Nb_2_O_5_ microcolumns.

Conventional Transmission Electron Microscope (TEM) images (Figure 2a,c,d) show that microcolumns have thicknesses ranging from 50 to 610 nm and that there is no variation in the thickness along the length of microcolumns. The selected Area Electron Diffraction (SAED) pattern (Figure 2b) confirms the pseudohexagonal crystal structure characteristic of Nb_2_O_5_ determined by XRD. In Figure 2d and Figure S1, the contrast inside the microcolumn with small bright areas (examples indicated with red arrows) surrounded by darker areas demonstrates that there is some porosity in the structure. High-Resolution TEM (HRTEM) images (Figure 2e,f) evidence the fact that the microcolumns contain grains. The boundary between such grains, evidenced by a red dotted line, is illustrated in Figure 2e. HRTEM also proves that the pores are amorphous areas between grains like the one delimited with a red line in Figure 2f. The diameter of pores ranges from 3 to 8 nm. EDX spectrum (Figure S2) confirms the presence in the sample of Nb and O besides the signals of C and Cu originating from TEM grid.

### 3.2. Gas Sensing

**Figure 3:**
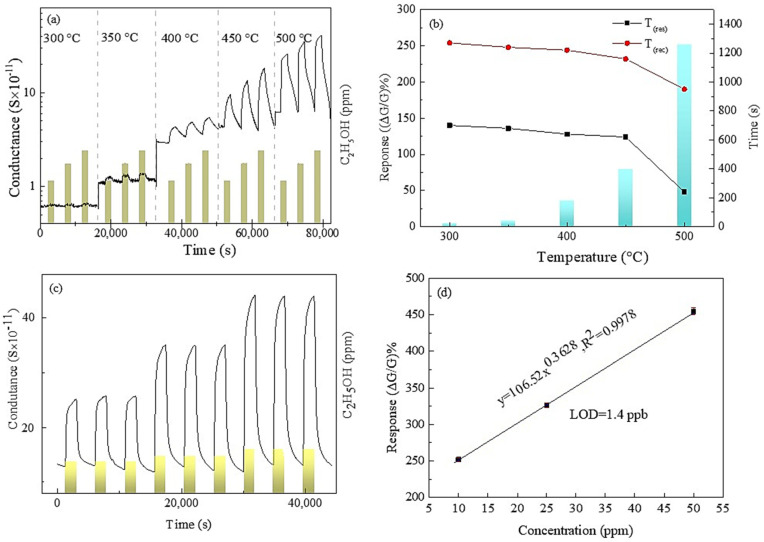
Gas sensing functionality of the Nb_2_O_5_ microcolumns; (**a**) dynamic response to ethanol at 300–500 °C; (**b**) response value, response/recovery times; (**c**) repeatability of the sensors signal at 500 °C; (**d**) modified power fitting.

Figure 3a displays the dynamic response of the chemiresistor ethanol sensors to 10, 25, and 50 ppm ethanol in the air. All the sensors show rising conductance upon exposure to ethanol. However, the sensor response was insignificant when the temperature was below 350 °C (Figure S3). This is due to the adsorption or reduction reaction of ethanol on the Nb_2_O_5_, releasing trapped electrons and increasing electron density. The increase in conductance confirms the n-type nature of the prepared Nb_2_O_5_ microcolumns, with positive electrons as the dominant charge carrier. Additionally, the sensors showed clear recovery after ethanol was cleared from the test chamber. The sensor response (ΔG/G%) and response and recovery times (time to reach 90% change in response during full response and recovery) were used to evaluate sensor performance. Figure 3b shows that higher sensor response and faster response and recovery times were achieved when the sensors were operating at 500 °C in dry air. 

The working temperature plays a crucial role in the response of sensors to ethanol. The temperature affects the sensing reaction by providing thermal energy that facilitates the reaction, which leads to an increased response to ethanol. For example, a study by Zhou et al. showed that an increase in working temperature leads to an increase in the response of sensors to ethanol [31]. Additionally, the temperature affects the amount of adsorbed ethanol molecules and oxygen species on the surface of the sensors, in which a higher working temperature leads to an increase in the amount of adsorbed ethanol molecules, which results in a higher response to ethanol. The study also showed that a higher amount of adsorbed ethanol molecules and oxygen species with increasing working temperature could affect the sensing response, which is temperature dependent. The highest response (ΔG/G%) was 251.23 with a response/recovery time of 240 s and 950 s, respectively, for detecting 10 ppm ethanol. Besides, it may be noted that the system needs 5 min to change its 1L environment with a flow rate of 200 sccm. Additionally, the response and recovery time was found to decrease with the increase in the operating temperature. It showed complete recovery and outstanding repeatability at optimal temperature (Figure 3c), making it ideal for practical use, as it eliminates baseline drift issues. Table 1 summarizes the comparison of ethanol sensors. However, operating a Nb_2_O_5_ sensor at 500 °C poses several challenges, including ensuring material stability, the reliability of electrical contacts, and the necessity of proper packaging and encapsulation to maintain the sensor’s performance and longevity.

**Figure 4:**
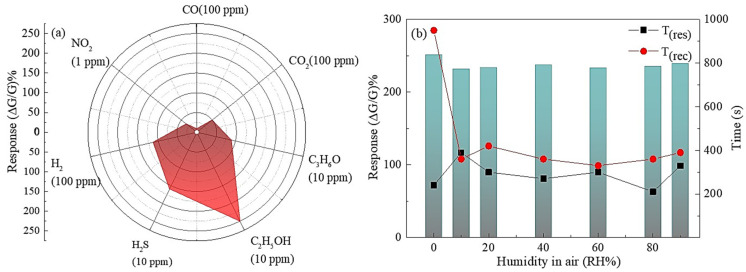
(**a**) Selectivity of the Nb_2_O_5_ sensors towards C_2_H_5_OH (10 ppm) compared to CO (100 ppm), CO_2_ (100 ppm), NO_2_ (1 ppm), H_2_ (100 ppm), H_2_S (10 ppm), and C_2_H_5_OH (10 ppm) at 500 °C in dry air; (**b**) humidity-dependent response, response/recovery time towards 10 ppm C_2_H_5_OH.

The sensor performance was further investigated, covering aspects such as selectivity, long-term stability (Figure S4), detection limit (LOD), and stability in humidity. The sensor response shows a linear relationship with ethanol concentration (Figure 3d) with a power-fitting (y = a·x^b^) correlation coefficient (R^2^) of over 0.99, meaning the experimental data fit well with a linear model. The LOD was determined using a 10% method, revealing the sensor’s capability to detect ethanol at 1.4 ppb when operating at 500 °C. Gas selectivity stands as a pivotal facet within gas sensor technology, especially in scenarios where discerning specific gases within a mixture holds paramount importance. The attainment of improved selectivity serves as a basis for ensuring the reliability and precision of gas sensors across diverse domains encompassing environmental surveillance, industrial safety protocols, and healthcare applications. In this study, we thoroughly assessed the performance of fabricated Nb_2_O_5_ sensors across a spectrum of gases, including CO, CO_2_, NO_2_, H_2_, H_2_S, and C_3_H_6_, alongside C_2_H_5_OH, at the optimal operational temperature of 500 °C. Remarkably, the sensors exhibited higher selectivity in ethanol detection, evidenced by a selectivity of 251.53%, when contrasted with CO (9.04%), CO_2_ (49.65%), NO_2_ (34.01%), H_2_ (113.55%), H_2_S (159.79%), and C_3_H_6_O (89.8%) at the lowest tested concentration, as depicted in Figure 4a.

The impact of relative humidity on the sensor’s performance to ethanol was investigated at different humidity levels (0, 10, 20, 40, 60, 80 and 90 RH%). Figure 4b displays the response and response/recovery times of the sensors to 10 ppm ethanol at 500 °C in varying humidity. No substantial change in response was observed with increasing humidity, but the baseline conductance decreased by 41% with 10% RH (as seen in Figure S5). Even with 90% RH, the baseline conductance remained unchanged from the baseline value with 10% RH. Usually, water molecules are absorbed on the MOXs by two means: physisorption (at higher RH%) and chemisorption (at lower RH%), which hinder the gas sensing functionality as well as the baseline conductance [32]. In this context, resistance change is caused by hydroxyl ions and mobile protons generated from the dissociation and adsorption of water molecules on the MOXs surface (active sites). Hydroxyl ions bond with metal cations while mobile protons (hydrogen ions) attach to oxygen at the MOX surface, creating additional hydroxyl ions. This process can impact the electrical conductance baseline based on the amount of hydroxyl ions and oxygen molecules present [33]. High humidity causes water molecules to absorb by transferring protons (H^+^) between incoming water and forming H_3_O^+^ at the MOX surface, leading to fluctuating electrical conductance. Our study found no significant decrease in baseline conductance compared to low humidity, which may be due to the high operating temperature. Despite changes in humidity, the response time remained constant, but the recovery time decreased by 59% compared to dry conditions. The quick recovery time of sensors in the presence of humidity may be due to the formation of C_2_H_5_OH_2_^+^·(H_2_O) with a low ionic diffusion coefficient [9,34]. 

**Table 1:** The comparison of ethanol-sensing performances of MOXs.

Materials	Methods	Temp. (°C)	Ethanol (ppm)	Resp.	Ref.
Au/SnO_2_	Hydrothermal	340	100	18.0	[35]
ZnO Nanowires	Oxidation	240	100	5.0	[36]
NiO/ZnO	VLS	400	50	6.7	[37]
NiO nanowires	VLS	400	50	2.9	[37]
Nb_2_O_5_-TiO_2_ nanofibers	Electrospinning	250	500	21.6	[9]
CuO-Fe_2_O_3_ hollow spheres	Template method	380	500	17.5	[38]
Nb_2_O_5_ microcolumns	Hydrothermal	500	10	2.51	This Work

### 3.3. Ethanol Sensing Mechanism

Nb_2_O_5_ is widely recognized as a wide-band n-type semiconductor, with conductivity affected by the surface depletion layer, a characteristic of surface-controlled sensing materials. Figure 4a indicates that the highest response among tested gases was seen with ethanol, making it crucial to examine the gas-sensing properties of the Nb_2_O_5_ sensors specifically concerning this gas for practical purposes. The detection mechanism is typical for chemoresistive gas sensors, as it involves the presence of oxygen forms (O^2−^, O^−^ or O_2_^−^) adsorbed on the Nb_2_O_5_ surface, which vary depending on the detection temperature [39,40]. The presence of oxygen species on Nb_2_O_5_ creates an electron depletion layer (EDL) on the surface by trapping electrons in the Nb_2_O_5_ conduction band during the reduction of O_2_-O^−^. However, it is crucial to understand the correct oxygen species that may be absorbed on the Nb_2_O_5_ surface to complete the discussion.

The relationship between gas concentration (C_g_) and response (S_g_) is widely recognized [39].

(4) $$S_{g}=1+a\cdot C_{g}^{a}$$

where the perfector and surface species charge parameters are represented by a and b, respectively. The value of b equals 0.5 or 1, indicating the absorbed oxygen ions on the surface to be either O^2−^ or O^−^ [40]. 

By taking the logarithm on either side of Equation (4), we can gain another equation that can be written as

(S_g_ − 1) = log(a) + b·log(C_g_) (5)

**Figure 5:**
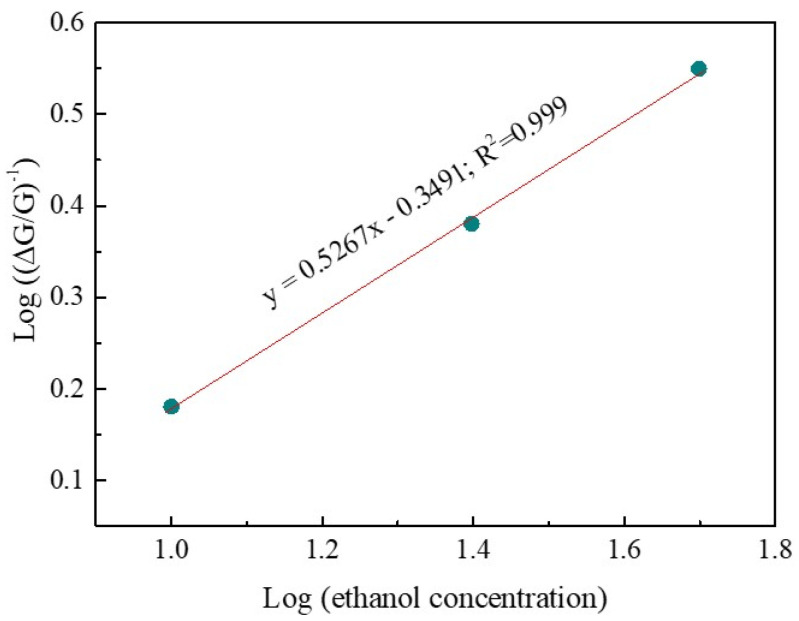
The plot of log(S_g_ − 1) vs. log(C_g_).

Figure 5 displays a log(S_g_ − 1) vs. log(C_g_) plot. The fit is linear, with a correlation coefficient exceeding 0.999. Thus, our study concludes that the dominant oxygen species is O^2−^. This leads to a redox reaction on the Nb_2_O_5_ surface between O^2−^ and C_2_H_5_OH upon exposure to C_2_H_5_OH, resulting in oxidation as follows [38].

C_2_H_5_OH (ads) + 6O^2−^ (ads) → 3H_2_O (g) + 2CO_2_ (g) + 12e^−^ (6)

As a result, when C_2_H_5_OH interacts with the oxygen species, the release of trapped electrons back to the conduction band of Nb_2_O_5_ causes an increase in conductance.

## 4. Conclusions

In conclusion, the synthesis of pseudohexagonal Nb_2_O_5_ microcolumns through a cost-effective hydrothermal process, followed by calcination, has yielded promising results for the development of high-performance conductometry sensors. Raman spectroscopy, HRTEM, SAED, and XRD confirmed the pseudohexagonal crystal lattice structure of Nb_2_O_5_, with thickness ranging from 50 to 610 nm and with pores ranging from 3 to 8 nm. 

The sensors exhibited outstanding selectivity for C_2_H_5_OH, even at low concentrations (2.51/10 ppm C_2_H_5_OH), surpassing their responsiveness to various other tested gases. Furthermore, the optimal operational temperature for this selectivity was found to be 500 °C in a dry air environment. Notably, the fabricated sensors demonstrated remarkable repeatability over four cycles and proved to be highly stable to humidity, even when exposed to 90% relative humidity. These excellent gas sensing properties can be attributed to the nonporous nature of the Nb_2_O_5_ microcolumns and the elevated operating temperature, making them promising candidates for innovative solutions in various industries, including environmental monitoring and industrial safety sensors.

## Data Availability

The data presented in this study are available on request from the corresponding author.

## Supplementary Materials

The following supporting information can be downloaded at: https://www.mdpi.com/article/10.3390/s24061851/s1, Figure S1: Magnified TEM images of Figure 2d; Figure S2: EDX spectrum of Nb_2_O_5_ microcolumns confirming the presence of Nb and O; Figure S3: Nb_2_O_5_ microcolumns sensor response to 10 ppm ethanol at different operating temperatures (300–500 °C); Figure S4: Long term stability of the Nb_2_O_5_ microcolumns sensor response to 10 ppm ethanol at 500 °C; Figure S5: Nb_2_O_5_ microcolumns sensor dynamic response to 10 ppm ethanol at different humidity levels when operating at optimum working temperatures (500 °C).
