# Supplementary material for: Nb2O5 Microcolumns for Ethanol Sensing
Source: Sensors (Basel). 2024 Mar 14;24(6):1851. doi: 10.3390/s24061851 (PMC10975242; doi:10.3390/s24061851)
Supplement: Supplementary file 1 [file sensors-24-01851-s001.zip › sensors-2890379-supplementary.pdf]

Nb_2O_5 MICROCOLUMNS FOR ETHANOL SENSING

Gayan C.W. Kumarage [1,2], Shasika A. Panamaldeniya [3,4], Valentin A. Maraloiu [5], Buddhika S. Dassanayake [4,] Nanda Gunawardhana [6,] and Elisabetta Comini [1],*

[1] SENSOR Lab, Department of Information Engineering, University of Brescia, Via Valotti 9, 25133 Brescia, Italy; g.wadumasthree@unibs.it, elisabetta.comini@unibs.it
[2] Department of Physics and Electronics, Faculty of Science, University of Kelaniya, Dalugama, 11600, Kelaniya, Sri Lanka; ckumarage@kln.ac.lk
[3] Postgraduate Institute of Science, University of Peradeniya, Peradeniya 20400, Sri Lanka
[4] Department of Physics, Faculty of Science, University of Peradeniya, Peradeniya 20400, Sri Lanka
[5] Laboratory of Atomic Structures and Defects in Advanced Materials, National Institute of Materials Physics, Atomistilor str. 405 A, Bucharest-Magurele, Romania; maraloiu@infim.ro
[6] Research and International Affairs, Sri Lanka Technological Campus, Padukka, 10500, Sri Lanka
***** Correspondence: elisabetta.comini@unibs.it

Supplementary information

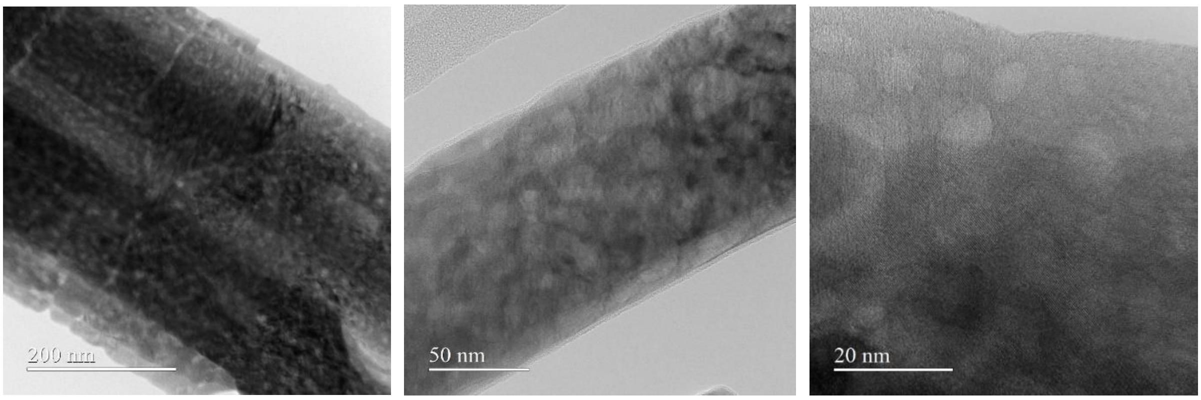

Figure S1. Magnified TEM images of Figure 2 (**d**).

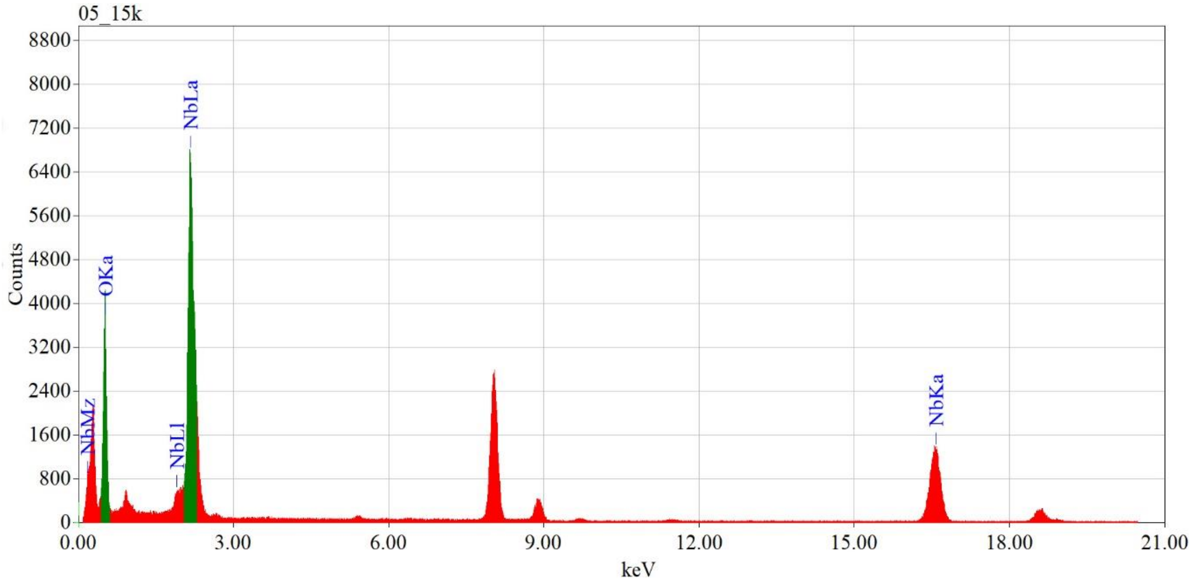

Figure S2. EDX spectrum of Nb_2O_5 microcolumns confirming the presence of Nb and O.

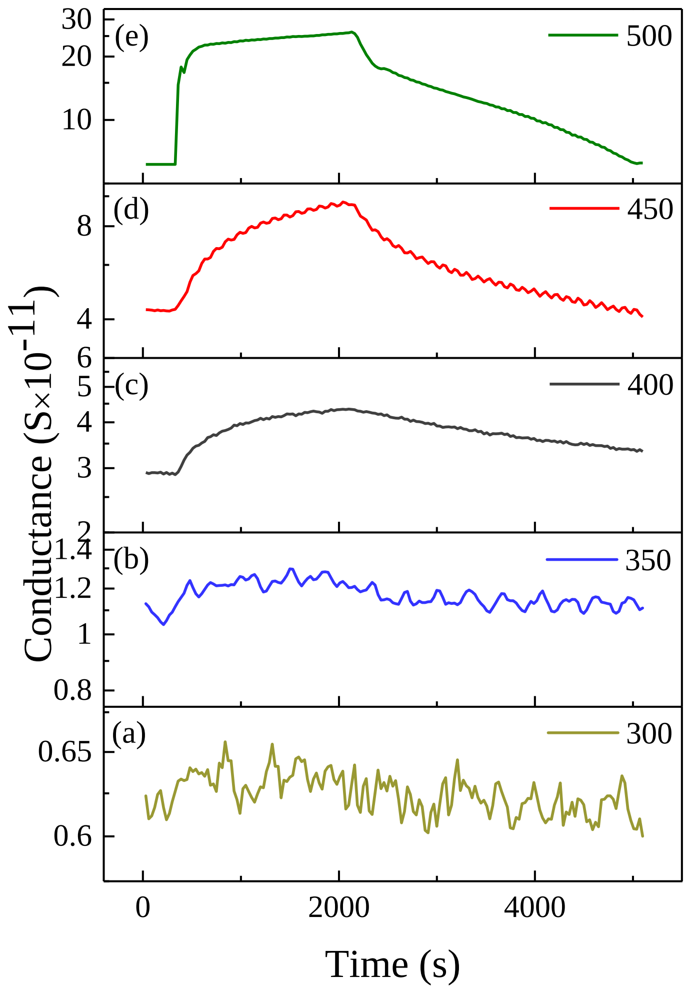

Figure S3. Nb_2O_5 microcolumns sensor response to 10 ppm ethanol at different operating temperatures (300-500 °C).

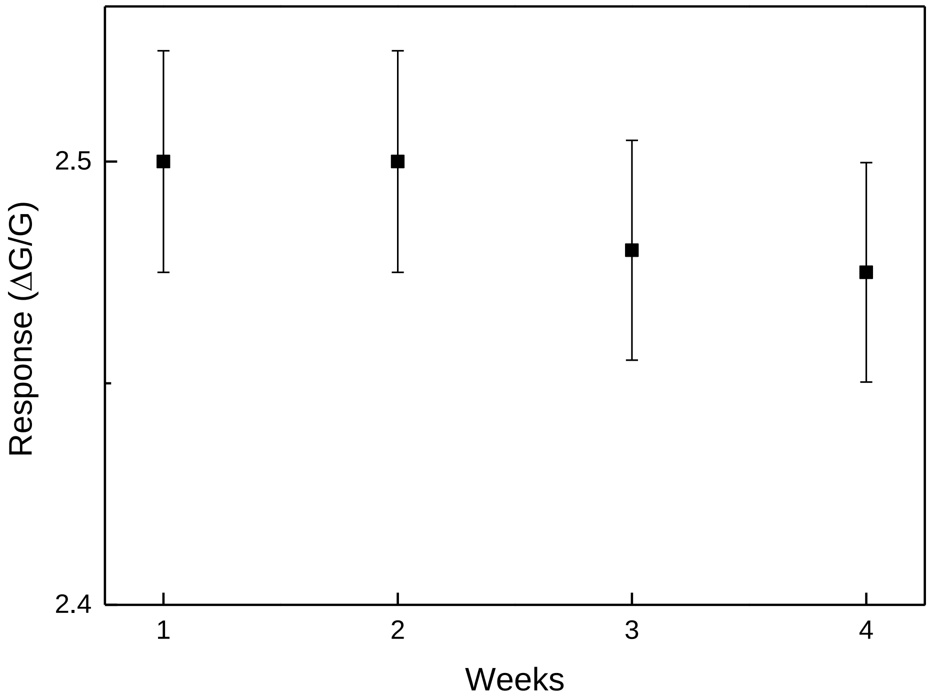

Figure S4. Long term stability of the Nb_2O_5 microcolumns sensor response to 10 ppm ethanol at 500 °C.

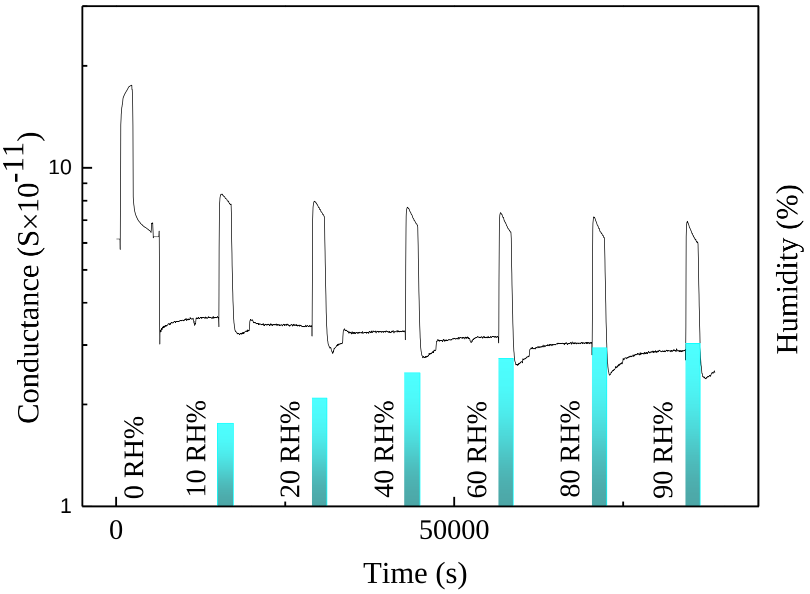

Figure S5. Nb_2O_5 microcolumns sensor dynamic response to 10 ppm ethanol at different humidity levels when operating at optimum working temperatures (500 °C).
